# Postoperative T1 tilt is a risk factor for postoperative distal adding-on in Lenke type 1 adolescent idiopathic scoliosis: A preliminary report

**DOI:** 10.1097/MD.0000000000019983

**Published:** 2020-05-22

**Authors:** Yusuke Sakai, Shota Takenaka, Takahiro Makino, Hideki Yoshikawa, Takashi Kaito

**Affiliations:** Department of Orthopaedic Surgery, Osaka University Graduate School of Medicine, 2-2 Yamadaoka, Suita, Osaka 565-0871, Japan.

**Keywords:** adolescent idiopathic scoliosis, distal adding-on, Lenke type 1, selective thoracic fusion, T1 tilt

## Abstract

Retrospective comparable study.

Postoperative loss of correction, which is referred to as the distal adding-on phenomenon, sometimes occurs during the postoperative course in Lenke type 1 adolescent idiopathic scoliosis (AIS). Selection of the lowest instrumented vertebra (LIV) has been reported to be one of the significant factors for preventing distal adding-on. However, proximal parameters, such as the Cobb angle of the proximal thoracic (PT) curve, radiographic shoulder height, and T1 tilt, were rarely described in previous reports. This study aimed to identify the risk factors for postoperative distal adding-on, including proximal radiographic parameters, in Lenke type 1 AIS.

Preoperative and postoperative radiographs of 34 consecutive patients with Lenke type 1 curve who underwent selective thoracic fusion were analyzed. The patients were divided into an adding-on group and a no-adding-on group according to the presence of adding-on at a 2-year follow-up. The 2 groups were compared with regard to age at surgery, Lenke lumbar modifier, Risser grade, instrumentation type, and radiographic parameters.

Distal adding-on was noted in 10 patients (29%). The adding-on group had significant variables including preoperative larger PT Cobb angle (*P* = .002), larger main thoracic (MT) flexibility (*P* = .006), smaller thoracolumbar (TL) Cobb angle (*P* = .012), larger LIV shift (*P* < .001), larger T1 tilt (*P* = .001), postoperative larger PT Cobb angle (*P* = .012), smaller MT Cobb angle (*P* = .016), smaller TL Cobb angle (*P* < .001), larger PT–MT mismatch (*P* < .001), larger LIV shift (*P* = .026), and larger T1 tilt (*P* = .006) when compared with the findings in the no-adding-on group. Postoperative T1 tilt was significantly correlated with PT–MT mismatch.

Our findings suggest that not only the LIV but also proximal parameters, including T1 tilt and PT–MT mismatch, are associated with postoperative distal adding-on in Lenke type 1 AIS. Strategies to reduce postoperative T1 tilt and PT–MT mismatch are required to prevent distal adding-on.

## 1 Introduction

Selective thoracic fusion surgery for Lenke type 1 adolescent idiopathic scoliosis (AIS) shows good clinical and radiographic results.^[1,2]^ However, some patients experience postoperative loss of correction, which is referred to as the distal adding-on phenomenon.^[3]^ Selection of the lowest instrumented vertebra (LIV) has been reported as one of the significant factors for preventing distal adding-on.^[3–5]^ Several radiographic variables, such as the neutral vertebra,^[6]^ the last touching vertebra,^[7]^ and intervertebral mobility in lateral bending, have been advocated for determining the LIV.^[8]^ However, these approaches are insufficient to completely prevent distal adding-on.

Recently, the relationship between postoperative shoulder balance and distal adding-on in Lenke type 2 AIS was reported.^[9]^ Furthermore, in a case series of hemivertebra resection for congenital cervicothoracic scoliosis, it was suggested that distal adding-on was a compensatory mechanism for correcting the head position.^[10]^ We thought that proximal radiographic parameters, such as the Cobb angle of the proximal thoracic (PT) curve, radiographic shoulder height, and T1 tilt, can affect distal adding-on in not only Lenke type 2 AIS and congenital scoliosis but also Lenke type 1 AIS. However, proximal parameters were rarely described in previous reports on type 1 AIS. Therefore, the present study aimed to comprehensively identify the risk factors for postoperative distal adding-on, including proximal radiographic parameters, in Lenke type 1 AIS.

## 2 Materials and methods

This was a retrospective comparative study at a single center. This study was reviewed and approved by our institution's ethics committee, and patients were given an opportunity to opt out of the study.

### 2.1 Patient population

The study enrolled 34 consecutive patients who underwent selective thoracic fusion for Lenke type 1 AIS at our hospital from March 2012 to May 2017. All included patients were female and had a single thoracic curve (convex to the right). The minimum follow-up period was 2 years. The cases in which the LIV was selected cephalad to the apex of the lumbar curve were considered selective thoracic fusion.^[11,12]^ In most cases, the end vertebra was determined as the LIV. When the end vertebra was not touched by the center sacral vertical line (CSVL), the last touching vertebra, defined as the last cephalad vertebra touched by the CSVL, was selected as the LIV.^[4]^ The upper instrumented vertebra (UIV) was determined to be the end vertebra or 1 level proximal to the end vertebra at the discretion of the surgeon.

At a 2-year follow-up, the patients were classified into 2 groups (adding-on group and no-adding-on group) on the basis of whether the distal adding-on phenomenon was observed according to the definition proposed by Wang et al^[3]^ as follows: a progressive increase in the number of vertebrae included within the distal curve, with either an increase of more than 5 mm in the deviation of the first vertebra below the instrumentation from the CSVL or an increase of more than 5° in the angulation of the first disc below the instrumentation.

### 2.2 Data collection

**Figure 1:**
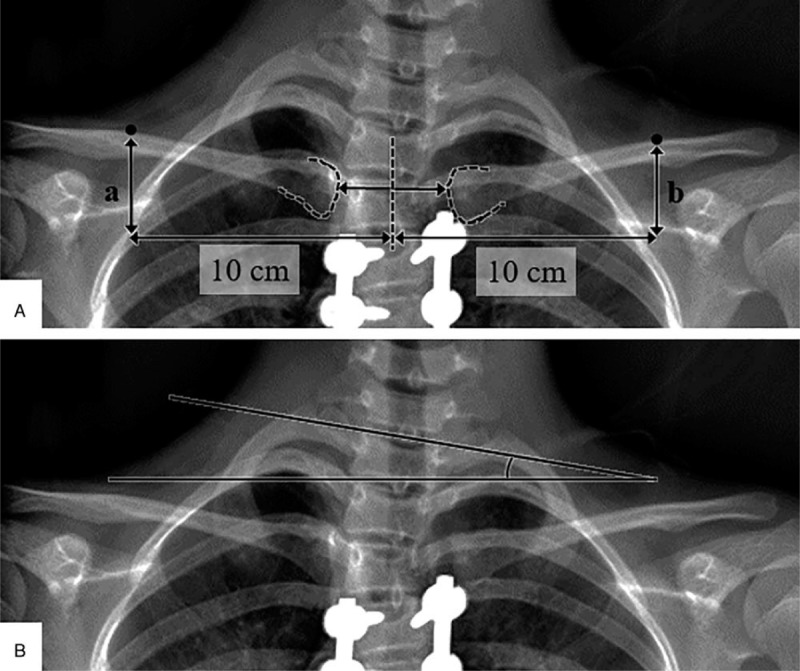
(A) Detailed measurement of radiographic shoulder height with calculation of the difference between points a and b (mm). (B) T1 tilt as the angulation between the T1 upper endplate and the horizontal line.

The 2 groups were compared with regard to age at surgery, Lenke lumbar modifier, Risser grade at surgery, instrumentation type (all-pedicle screw or hybrid construct), selection of UIV and LIV, and the following radiographic parameters in the preoperative and early postoperative periods: Cobb angle and flexibility of PT curve, main thoracic (MT) curve, and thoracolumbar (TL) curve, PT–MT mismatch (value obtained by subtracting the MT Cobb angle from the PT Cobb angle), correction rate of each curve, fusion mass Cobb angle (FMC), C7 shift from CSVL, apical translation, LIV shift from CSVL, radiographic shoulder height (RSH), and T1 tilt angle (Fig. 1). A positive value of the T1 tilt was defined as inclination to the right.

### 2.3 Surgical procedures

Of the 34 patients, 15 received the hybrid construct before 2014 and 19 received the all-pedicle screw construct later. In the hybrid construct group, after placement of hooks and screws, a rod contoured to the thoracic kyphosis was placed at the concave side. It was rotated by 90° for scoliosis correction and kyphosis creation. Scoliosis was further corrected using in situ benders. An underbent convex rod was placed, and segmental compression and distraction were performed. In the all-pedicle screw construct group, the surgical procedures for scoliosis correction were almost similar to those performed in the hybrid construct group. Direct vertebral derotation via pedicle screws placed on both sides was additionally performed.

### 2.4 Statistical analysis

The Mann–Whitney *U* test was used to compare continuous variables in demographic and radiographic data between the adding-on group and the no-adding-on group. Fisher's exact test was used to compare group differences with regard to categorical variables. Pearson's correlation coefficient was used to assess the correlations between T1 tilt and other radiographic parameters. All statistical analyses were performed using SPSS statistical software (v. 21.0; IBM Corp., Armonk, NY). A *P*-value <.05 was considered statistically significant.

## 3 Results

The distal adding-on phenomenon was observed in 10 of the 34 patients (29%) at a 2-year follow-up; however, no patient required additional operation. The adding-on group and the no-adding-on group were comparable with regard to the mean age at surgery, Lenke lumbar modifier, Risser grade, instrumentation type, UIV, and LIV (Table 1).

**Table 1:**
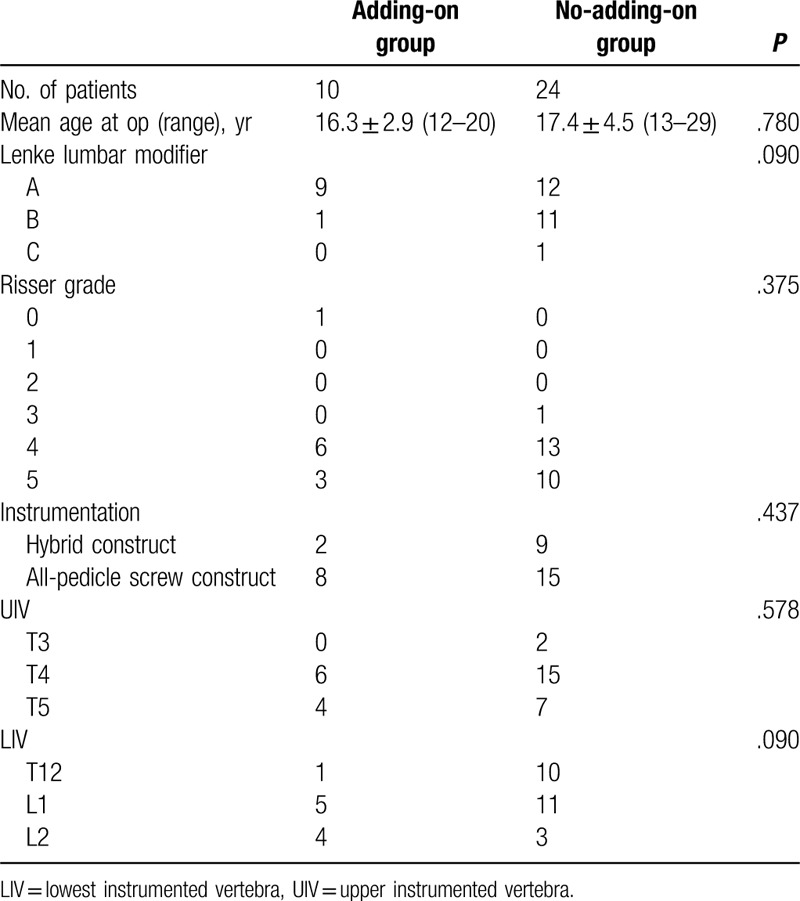
General information of patients.

In standing posteroanterior radiographs before surgery, the adding-on group had a larger PT Cobb angle (*P* = .002), larger MT flexibility (*P* = .006), smaller TL Cobb angle (*P* = .012), larger apical translation (*P* = .028), larger LIV shift (*P* < .001), and larger T1 tilt (*P* = .001) when compared with the findings in the no-adding-on group (Table 2).

**Table 2:**
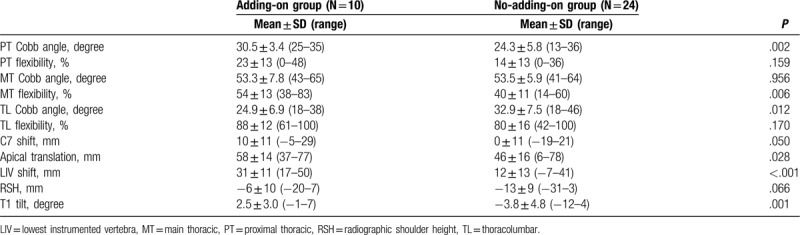
Preoperative radiographic parameters.

**Figure 2:**
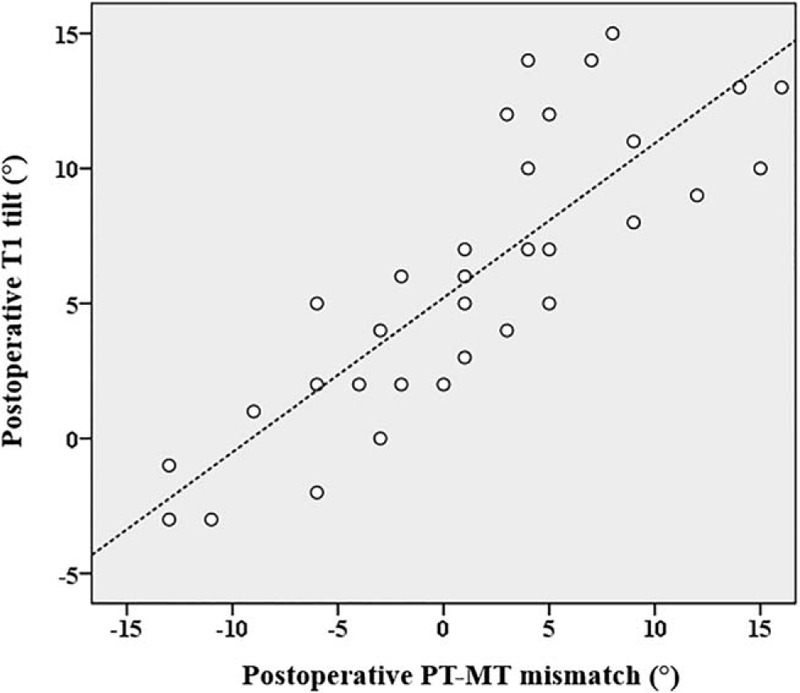
The correlation of postoperative T1 tilt with PT–MT mismatch. T1 tilt is strongly correlated with PT–MT mismatch (*r* = 0.835, *P* < .001). MT = main thoracic, PT = proximal thoracic.

In the early postoperative period, the adding-on group had a larger PT Cobb angle (*P* = .012), smaller MT Cobb angle (*P* = .016), smaller TL Cobb angle (*P* < .001), larger PT–MT mismatch (*P* < .001), smaller FMC (*P* = .012), larger LIV shift (*P* = .026), and larger T1 tilt (*P* = .006) when compared with the findings in the no-adding-on group (Table 3). The postoperative T1 tilt was significantly correlated with the preoperative PT Cobb angle (*r* = 0.762, *P* < .001) and PT–MT mismatch (*r* = 0.835, *P* < .001) (Table 4 and Fig. 2). There was no significant difference in RSH between the 2 groups both preoperatively and early postoperatively.

**Table 3:**
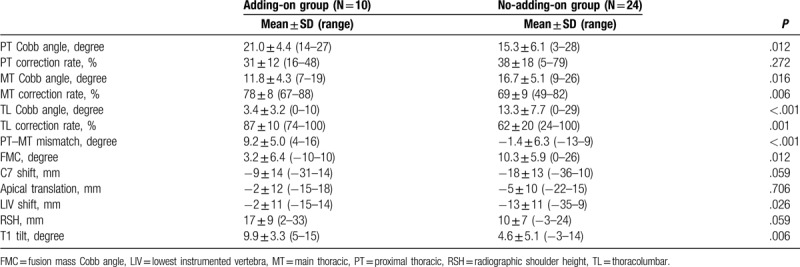
Postoperative radiographic parameters.

**Table 4:**
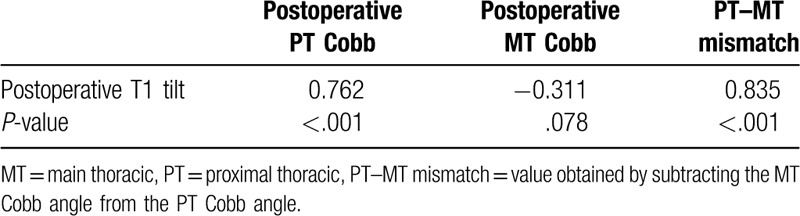
Pearson's correlation coefficients between postoperative T1 tilt and PT–MT mismatch.

**Figure 3:**
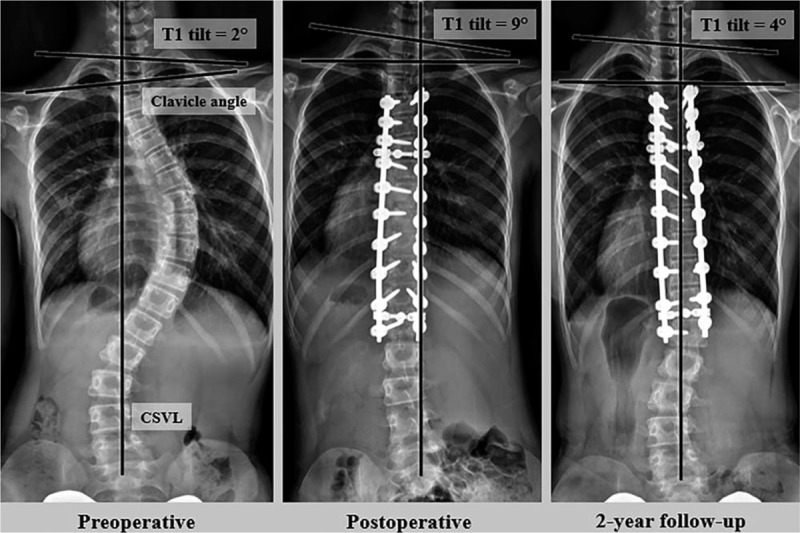
A 12-yr-old female patient. Although the postoperative shoulder balance is good, T1 tilt shows an increase from 2° to 9° after surgery. At a 2-yr follow-up, the distal adding-on is noted, and the T1 tilt shows a decrease to 4°.

The radiographs of a representative case are presented in Figure 3. A 12-year-old female patient underwent selective thoracic fusion from T4 to L1 with an all-pedicle screw construct. The preoperative PT, MT, and TL Cobb angles were 34°, 61°, and 38°, respectively, and the angles reduced to 20°, 8°, and 10°, respectively, early postoperatively. Although the postoperative shoulder balance was good, the T1 tilt increased from 2° before surgery to 9° after surgery. At a 2-year follow-up, the distal adding-on phenomenon was observed, and the T1 tilt decreased to 4°.

## 4 Discussion

Distal adding-on is a common complication in AIS patients after correction surgery, and it was first reported by Suk et al.^[13]^ The incidence of adding-on has been reported to be 2% to 51%.^[2–5]^ The cause of adding-on has been considered to be multifactorial, including LIV selection,^[2–5]^ lumbar vertebra rotation,^[14]^ apical translation of the MT curve,^[4]^ and open tri-radiate cartilage.^[15]^

In this study, distal adding-on was observed in 29% of the patients at a 2-year follow-up. Multiple parameters were identified as risk factors by comparing the adding-on group with the no-adding-on group. These parameters represent 3 categories (vertebral shift from CSVL, T1 tilt, and Cobb angle), and the categories are discussed below.

The first risk factor category of vertebral shift form CSVL includes preoperative apical translation, and both preoperative and postoperative LIV shift. Among these factors, LIV shift has been mentioned in many studies as a risk factor for distal adding-on, and LIV selection has especially attracted attention to prevent distal adding-on. Suk et al recommended a focus on the neutral vertebra when determining LIV.^[6]^ They observed an increased risk of adding-on when the LIV was proximal to the neutral vertebra by more than 2 vertebrae. Ni et al suggested LIV selection according to intervertebral mobility in preoperative side-bending radiographs.^[8]^ Xu et al showed the usefulness of touch classification.^[7]^ Our results are consistent with the findings of these previous reports mentioning that LIV deviation is one of the risk factors for distal adding-on. We believe that LIV selection is important for preventing distal adding-on. However, there is a dilemma that fusion range extension to the distal side can cause increased low back pain and lumbar stiffness.^[16]^

The second category is T1 tilt. In this study, it was demonstrated to be a risk factor both preoperatively and postoperatively. Proximal parameters as risk factors for distal adding-on have rarely been investigated in previous reports. With regard to Lenke type 2, there has been only 1 report in which postoperative shoulder balance was a risk factor for distal adding-on.^[9]^ Our study focused on Lenke type 1 and investigated the relationships between proximal parameters and distal adding-on. We found that the adding-on group tended to show shoulder imbalance inclined to the right postoperatively. However, statistically, T1 tilt rather than RSH was a risk factor for distal adding-on. Although it is uncertain why T1 tilt was related to distal adding-on, we hypothesized that adding-on occurred to compensate for the height difference between the eyes associated with torticollis. This compensation mechanism is supported by a recent study on hemivertebra resection for congenital cervicothoracic scoliosis. In this previous report, Chen et al mentioned that the postoperative head shift and clavicle angle decreased consistently with slight progression of the distal compensatory curve.^[10]^ We believe that this mechanism can explain our results of an early postoperative larger T1 tilt in the adding-on group, but further studies are needed to confirm the mechanism.

The third category of Cobb angle includes preoperative large MT flexibility, postoperative small MT Cobb angle, large PT–MT mismatch, small FMC, and both preoperative and postoperative large PT Cobb angles and small TL Cobb angles. It is noteworthy that MT correction was significantly greater in the adding-on group than in the no-adding-on group in this study. This difference may be due to a mismatch between good MT correction and a relatively large residual PT Cobb angle. A large PT–MT mismatch can lead to a postoperative large T1 tilt. In fact, the postoperative T1 tilt was significantly correlated with the preoperative PT Cobb angle and PT–MT mismatch. There have been no previous reports on this relationship between distal adding-on and PT–MT mismatch. Progress in instrumentation for spinal correction surgery, especially frequent use of pedicle screws, has made it possible to achieve good AIS correction. However, it is necessary to consider that good MT curve correction may have a negative effect on the risk of distal adding-on.

According to these risk factor categories, it is important to clarify which aspect should be focused on when performing corrective surgery for Lenke type 1 AIS. It appears obvious that LIV selection is important, as demonstrated in previous reports. Furthermore, strategies to reduce postoperative T1 tilt and PT–MT mismatch may be important to prevent distal adding-on. Specifically, we should consider an option to extend the fusion range to the proximal side when a large PT Cobb angle or large MT flexibility is observed in preoperative radiographs. In addition, during surgery, we should avoid excessive correction of the MT curve without correction of the PT curve. Consideration of T1 tilt and PT–MT mismatch will help reduce the incidence of distal adding-on in Lenke 1 AIS, leading to improved surgical strategies.

The present study has some limitations. First, this observational study involved retrospective data collection with inconsistency of the surgical technique for deformity correction. However, it was found that differences in instrumentation (all-pedicle screw or hybrid construct) did not affect distal adding-on. Second, this study did not consider clinical outcomes and only considered radiographic outcomes. As no additional surgery was required during a 2-year follow-up, it can be considered that no major clinical problem was present. However, we should confirm whether adding-on is a clinical problem or a minor complication only in radiographs. Third, the number of cases was small to make a solid conclusion, as this study was conducted as a preliminary clinical study. Further studies with a large sample size should follow this preliminary report. The radiographic parameters identified as risk factors in this study may confound each other. Therefore, multivariate analysis will be useful to confirm the relationships between the parameters and adding-on. Although these limitations are important, to our knowledge, this is the first study to investigate the associations of T1 tilt and PT–MT mismatch with distal adding-on in Lenke type 1 AIS.

## 5 Conclusions

The risk factors for postoperative distal adding-on in Lenke type 1 AIS were comprehensively identified in this study. The risk factors were classified into the following 3 categories:

(1) vertebral shift form CSVL (apical translation and LIV shift to the right),
(2) T1 tilt, and
(3) Cobb angle (large PT and small MT [PT–MT mismatch]).

T1 tilt and PT–MT mismatch were demonstrated for the first time as risk factors for distal adding-on. Strategies to reduce postoperative T1 tilt and PT–MT mismatch are required to prevent adding-on.

## Author contributions

**Conceptualization:** Yusuke Sakai, Shota Takenaka, Takashi Kaito.

**Data curation:** Yusuke Sakai, Shota Takenaka, Takahiro Makino.

**Formal analysis:** Yusuke Sakai, Shota Takenaka.

**Investigation:** Yusuke Sakai, Takahiro Makino.

**Methodology:** Yusuke Sakai, Shota Takenaka.

**Supervision:** Hideki Yoshikawa, Takashi Kaito.

**Writing – original draft:** Yusuke Sakai.

**Writing – review & editing:** Shota takenaka, Takashi Kaito.
